# Matrix Metallopeptidase 14: A Candidate Prognostic Biomarker for Diffuse Large B-Cell Lymphoma

**DOI:** 10.3389/fonc.2020.01520

**Published:** 2020-08-20

**Authors:** Chengliang Yin, Junyan Zhang, Ming Shen, Zhenyang Gu, Yan Li, Wanguo Xue, Jinlong Shi, Wenrong Huang

**Affiliations:** ^1^National Engineering Laboratory for Medical Big Data Application Technology, Chinese PLA General Hospital, Beijing, China; ^2^Medical Big Data Research Center, Chinese PLA General Hospital, Beijing, China; ^3^Translational Medicine Laboratory, Chinese PLA General Hospital, Beijing, China; ^4^Department of Hematology, Chinese PLA General Hospital, Beijing, China; ^5^Department of Biomedical Engineering, Chinese PLA General Hospital, Beijing, China

**Keywords:** matrix metallopeptidase 14, diffuse large B-cell lymphoma, immune cell infiltration, prognosis, biomarker

## Abstract

**Background:**

Matrix metallopeptidase 14 (MMP14) is an important gene in the regulation of T-cell function. However, the correlation between MMP14 expression, prognosis, and immune cell infiltration in diffuse large B-cell lymphoma (DLBCL) remains unclear.

**Methods:**

We investigated the influence of MMP14 on clinical prognosis using data obtained from three Gene Expression Omnibus (GEO) database sets (GSE98588, GSE10846, and GSE4475). The expression of MMP14 was analyzed using the Gene Expression Profiling Interactive Analysis (GEPIA). The correlation between MMP14 and immune cell infiltration was investigated using the Cell-type Identification By Estimating Relative Subsets Of RNA Transcripts (CIBERSORT) and Tumor Immune Estimation Resource (TIMER) tools. In addition, the correlation between MMP14 expression and immune gene markers was analyzed by TIMER and GEPIA.

**Results:**

MMP14 expression positively correlated with favorable progression-free survival (PFS; GSE98588, *P* = 0.02) and overall survival (OS; GSE98588, *P* = 0.003; GSE10846, *P* = 5.517e-05; and GSE4475, *P* = 9.85e-04). Moreover, MMP14 expression was higher in DLBCL tumors than in normal tissues. Regarding clinical characteristics, high MMP14 expression was found to be correlated with race. MMP14 expression was also correlated with immune cell infiltration and had a remarkable correlation with various immune marker sets. It was found that M0 macrophages were the immune cells most related to survival, decreasing with the increase in Ann Arbor clinical stage. The results especially showed that MMP14 was a prognostic biomarker and related to the macrophages M0.

**Conclusion:**

The results suggest that MMP14 is a novel prognostic molecular marker for DLBCL and is related to the immune cell infiltration, especially related to the macrophages M0. Our study provides insights for understanding the potential roles of MMP14 in tumor immunology and its suitability as a prognosis biomarker in DLBCL.

## Introduction

Diffuse large B-cell lymphoma (DLBCL) is the clinically and genetically heterogeneous group comprising malignant proliferations of large lymphoid B cells, according to the definition by the World Health Organization (WHO) classification (1, 2). Although immunochemotherapy-based combination therapy (R-CHOP; rituximab plus cyclophosphamide, doxorubicin, vincristine, and prednisone) and better supportive care have improved the survival rate, the prognosis of refractory and relapsed patients remains poor (3). Thus, there is an urgent need to identify novel potential prognostic biomarkers and to investigate the mechanisms of immune involvement in tumorigenesis and tumor progression in the context of DLBCL.

Matrix metallopeptidase 14 (MMP14) is a membrane anchored MMP, which cleaves other functional proteins such as interleukin 8 and pro-tumor necrosis factor to maintain the tumor microenvironment (4–6). Several studies reported a role for MMP14 in susceptibility, pathological development, microenvironmental regulation, and prognosis of hepatocellular carcinoma (7), ovarian carcinoma (5), breast cancer (8), epithelial skin cancers (9), colorectal cancer (10), and gastric cancer (11). Thus, enhancing the immune response against the tumor could benefit patients affected with DLBCL, improving their survival (12, 13). However, there is only little research about this field, and feasible immune biomarkers for the prediction of DLBCL patients’ prognosis are lacking. Moreover, the role of MMP14 in the clinical prognosis and immune cell infiltration in DLBCL remains unclear. In this study, we investigate potential new prognostic markers for DLBCL using a bioinformatics approach with data already available in public datasets.

## Materials and Methods

### Gene Expression Omnibus Databases Analysis

Gene Expression Omnibus (GEO; http://www.ncbi.nlm.nih.gov/geo/) is an international public repository containing high-throughput microarray and next-generation sequencing functional genomic data sets (14). All genes from two independent DLBCL GEO cohorts [GSE10846, 420 samples (15); GSE98588, 137 samples (2)] were analyzed. According to the media based on each gene expression value, we subdivided DLBCL patients into low- and high-expression groups. Overall survival analyses (OS) in correlation to all genes were performed to identify overlapping genes between the two cohorts for the two different groups. The statistical cut-off value was *P* < 0.001. The overlap was evaluated using a Venn diagram^1^. Gene Ontology (GO) is a community-based bioinformatics resource containing information about a gene product using function-based categories (16). The Kyoto Encyclopedia of Genes and Genomes (KEGG) is a knowledge database for the systematic analysis of gene functions, connecting genomic information with higher-order functional information (17). Functional enrichment of overlapping genes based on GO and KEGG annotations (top five modules) was performed using the Metascape tool (18). The consistent candidate prognosis genes were selected among overlapping genes with a potential correlation to DLBCL, as assessed using the Oncomine database^2^ and literature search.

### The Expression of Matrix Metallopeptidase 14 and Survival Analysis

The Gene Expression Profiling Interactive Analysis (GEPIA; http://gepia.cancer-pku.cn/) is a web-based analysis tool based on The Cancer Genome Atlas (TCGA) and the Genotype-Tissue Expression (GTEx) data (19). GEPIA was used to obtain the MMP14 expression data in cancer and normal tissues (19). The correlation between MMP14 expression and the Kaplan–Meier survival analysis progression-free survival (PFS, GSE98588; OS, GSE98588, and GSE10846) in DLBCL cancer was analyzed, which was further validated in another GEO database [OS, GSE4475 (20)].

### Diffuse Large B-Cell Lymphoma Data in the Cancer Genome Atlas Analysis

The Cancer Genome Atlas is a public-funded project that seeks to catalog and discover major cancer-causing genomic alterations to create a comprehensive “atlas” of cancer genomic profiles (21). RNA-Seq data of 48 patients affected with DLBCL were downloaded from TCGA. Datasets also contained clinical data such as Ann Arbor clinical stage, gender, race, body mass index (BMI), age, and survival time. Then the relationship between MMP14 expression and Ann Arbor clinical stage, gender, race, BMI, and age was investigated. We also used TCGA data to perform gene set enrichment analysis (GSEA), a statistical method to determine if predefined sets of genes are differentially expressed among selected samples (22). GSEA was performed on low- and high-expression groups, according to the median MMP14 expression values. GO terms and the KEGG pathways analyses were also performed to investigate possible novel biological functions of MMP14 in DLCBL. For this analysis, we selected the most highly enriched signaling pathways based on the normalized enrichment score (NES).

### CIBERSORT and TIMER Database Analysis

Cell-type Identification By Estimating Relative Subsets Of RNA Transcripts (CIBERSORT; http://cibersort.stanford.edu), a method for characterizing cell composition of complex tissues from their gene expression profiles (23), was used to obtain the abundance ratio matrix of 22 immune cell types using RNA-seq data from TCGA-DLBCL (23). The differential abundance of immune infiltrates was obtained comparing the distribution of immune cells in low- and high-MMP14 expression groups using the R software. Then a correlation analysis was performed using the 11 immune cell types whose presence was identified with CIBERSORT in the 48 RNA-seq samples.

Tumor Immune Estimation Resource (TIMER; https://cistrome.shinyapps.io/timer/) is a comprehensive resource for the systematic analysis of immune infiltrates. This repository includes 10,897 samples based on 32 cancer types obtained from TCGA (24). TIMER uses a published deconvolution statistical method to estimate the abundance of tumor-infiltrating immune cells (B cell, CD4 T cell, CD8 T cell, neutrophil, macrophage, and dendritic cell) from gene expression profiles (25). TIMER was used to measure the correlation between MMP14 expression and the abundance of six types of immune infiltrating cells. Tumor purity was used to reduce bias for this analysis (26). In addition, we investigated the correlation between MMP14 expression and various gene markers (27–30) of tumor-infiltrating immune cells using correlation modules. Moreover, GEPIA was used to further validate significantly correlated genes identified with TIMER.

### Clinical Relationship With the Immune Cell Most Related to Survival

We performed Kaplan–Meier analysis to measure the OS of the 48 patients obtained from TCGA based on the abundance ratio of the 11 identified immune cell types, for which the cut-off level was set at the median value. We also identified the immune cell most related to survival based on the results of the Kaplan–Meier survival analysis. Moreover, we investigated the relationship between the immune cell that was most related to survival and the clinical features (Ann Arbor clinical stage, gender, race, BMI, and age) in the 48 samples. The statistical significance between the two variates was calculated using *t*-test for independent samples, whereas for more variates, one-way ANOVA was used. Furthermore, we performed a correlation analysis between low- and high-expressed gene groups selected according to the median abundance values of the immune cell that was most related to survival. Differentially expressed genes were identified using the R package edgeR, with the following parameters: fold change >1.5 (for up-regulated genes) or fold change <2/3 (for down-regulated genes), and *P* < 0.01. Metascape^3^ was used to perform GO and KEGG pathway enrichment analysis, which was used to predict the potential biological functions of the differentially expressed genes.

## Results

### The Selection of Matrix Metallopeptidase 14 Gene

**FIGURE 1:**
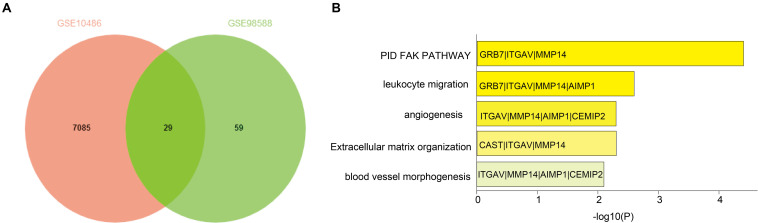
**(A)** Venn diagram of overlapping differentially expressed genes in GSE10846 and GSE98588 diffuse large B-cell lymphoma (DLBCL) datasets. **(B)** Functional enrichment of overlapping genes based on Gene Ontology (GO) and Kyoto Encyclopedia of Genes and Genomes (KEGG) analyses.

We identified 29 overlapping genes (Supplementary Table S1) in two independent DLBCL cohorts (GSE10846, 420 samples; GSE98588, 137 samples, *P* < 0.001) via a Venn diagram. These genes significantly correlated with OS based on the median gene expression values (Figure 1A). Interestingly, MMP14 was enriched in all the highest-ranked biological processes and KEGG pathways among the 29 overlapping genes as determined by Metascape analysis (Figure 1B). These potential prognostic genes were further analyzed for their presence in DLBCL cases using the Oncomine database and by the literature search. We then selected MMP14 as our candidate prognostic marker in DLBCL.

### Matrix Metallopeptidase 14 Expression Predicted Prognosis in Diffuse Large B-Cell Lymphoma

**FIGURE 2:**
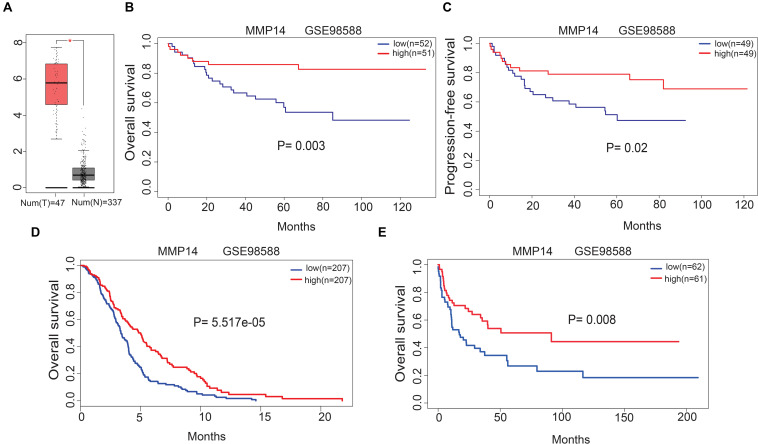
**(A)** Matrix metallopeptidase 14 (MMP14) expression levels in DLBCL tissues [Gene Expression Profiling Interactive Analysis (GEPIA) database]. T, tumor; N: normal tissue. **(B)** Correlation of MMP14 expression with overall survival (OS) in the GSE98588 dataset. **(C)** Correlation of MMP14 expression with progression-free survival (PFS) in the GSE98588 dataset. **(D)** Comparison of MMP14 expression with OS in the GSE10846 dataset. **(E)** Comparison of MMP14 expression with OS in the GSE4475 datasets (validation group).

Gene Expression Profiling Interactive Analysis uncovered that the MMP14 expression was higher in DLBCL tumors than in normal tissues (Figure 2A). We investigated the correlation of MMP14 expression with prognosis in different cohorts of DLBCL patients. Indeed, the high-MMP14 expression group was associated with favorable PFS (GSE98588, *P* = 0.02) and OS (GSE98588, *P* = 0.003; GSE10846, *P* = 5.517e-05; Figures 2B–D). Similarly, we also found that the overexpression of MMP14 is correlated to a favorable OS in the GSE4475 dataset (*P* = 0.008; Figure 2E). Therefore, our results suggest that MMP14 expression is associated with prognosis in DLBCL patients.

### Matrix Metallopeptidase 14 Expression Correlation With Clinical Characteristics and Predicted Biological Functions

To enhance the comprehension about the relevance of MMP14 expression in DLBCL cancer, we analyzed the relationship between MMP14 expression and clinical characteristics using the TCGA-DLBCL database. We found that MMP14 expression was significantly correlated with race, with White patients showing the highest MMP14 expression (*P* < 0.05), but was not significantly correlated to other clinical characteristics, such as tumor stage, gender, BMI, and age (Supplementary Table S2).

**FIGURE 3:**
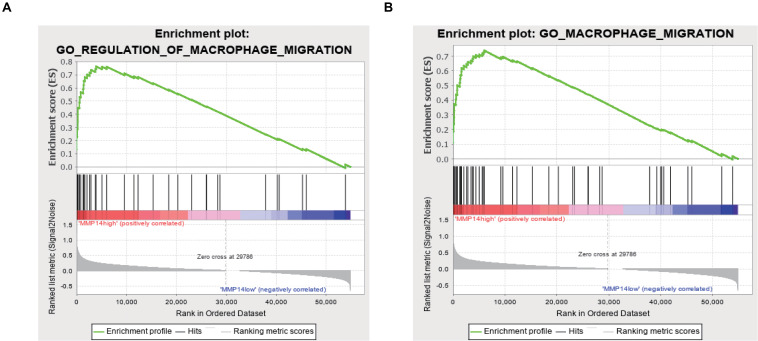
**(A)** GO Enrichment plot of the regulation of macrophage migration profile of the analyzed gene set. The enrichment score (ES) is shown and genes are ordered according to the rank-ordered list. **(B)** GO enrichment plot of the macrophage migration profile of the analyzed gene set. The enrichment score (ES) is shown, and genes are ordered according to the rank-ordered list.

To explore the potential MMP14 biological function, we then performed GO term and KEGG pathway analyses (Table 1 and Supplementary Table S3). GO annotation showed five positively correlated categories with high levels of MMP14: regulation of macrophage migration, macrophage migration, collagen metabolic process, iron ion homeostasis, and collagen catabolic process. The GO analysis also revealed five negatively correlated categories: nuclear-transcribed mRNA catabolic process, viral gene expression, nuclear-transcribed mRNA catabolic process nonsense-mediated decay, rRNA methylation, and translational initiation. The KEGG pathway analysis revealed the following five strongest positively correlated pathways with MMP14 expression: lysosome, glycosaminoglycan degradation, extracellular matrix receptor interaction, other glycan degradation, and complement and coagulation cascades. Conversely, the five pathways with the strongest negative correlation were RNA polymerase, ribosome, nucleotide excision repair, spliceosome, and base excision repair. These results suggest that pathways regulating macrophage migration and nuclear transcription were critically important in DLBCL patients and that macrophages seem to be strongly associated with MMP14 expression (Figures 3A,B).

**TABLE 1:** Most significantly correlated signaling pathways with matrix metallopeptidase 14 (MMP14) expression, based on their normalized enrichment score (NES) and *P-*value (top five modules).

	GO name	NES	NOM *p*-value	FDR *q*-value
Positive	Regulation of macrophage migration	2.248	0	9.46e-04
	2.246	0		4.73e-04
	2.246	0		3.15e-04
	Iron ion homeostasis	2.202	0	4.78e-04
	Collagen catabolic process	2.199	0	3.82e-04
Negative	Nuclear-transcribed mRNA catabolic process	−2.112	0	5.89e-023
	Viral gene expression	-2.031	0	1.19e-02
	Nuclear-transcribed mRNA catabolic process nonsense-mediated decay	−2.005	2.08e-03	1.39e-02
	rRNA methylation	−1.993	2.07e-03	1.48e-02
	Translational initiation	−1.993	4.23e-03	1.19e-02

	**KEGG name**	**NES**	**NOM *p*-value**	**FDR *q*-value**

Positive	Lysosome	2.246	0	0
	Glycosaminoglycan degradation	2.012	0	2.11e-02
	Ecm receptor interaction	1.990	0	2.00e-02
	Other glycan degradation	1.986	0	1.76e-02
	Complement and coagulation cascades	1.917	0	3.47e-02
Negative	RNA polymerase	−1.729	2.35e-02	2.00e-01
	Ribosome	−1.644	4.55e-02	2.27 e-01
	Nucleotide excision repair	−1.636	4.58e-02	1.63 e-01
	Spliceosome	−1.633	5.54e-02	1.24 e-01
	Base excision repair	−1.584	6.11e-02	1.46 e-01

### Matrix Metallopeptidase 14 Expression Is Correlated With Immune Cell Infiltration in Diffuse Large B-Cell Lymphoma

**FIGURE 4:**
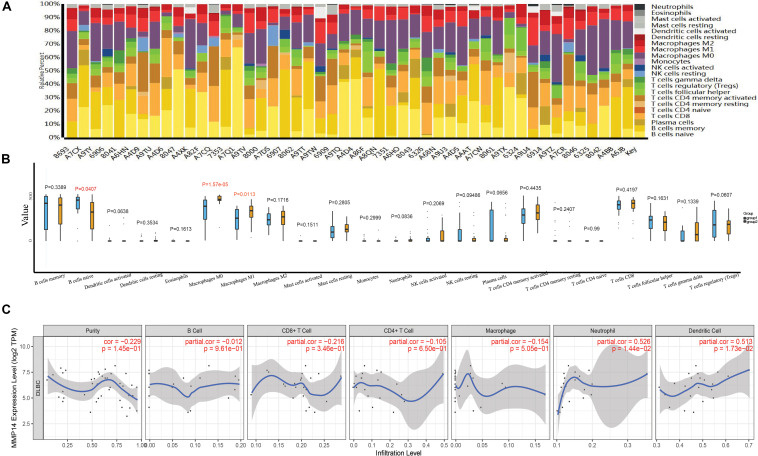
**(A)** Bar plots of the abundance ratio of 22 immune cell types in 48 The Cancer Genome Atlas (TCGA)-DLBCL samples. Each type is shown in a different color. **(B)** Boxplots showing the infiltration levels of 22 immune cell types in low- and high-MMP14 expression groups. Blue: low-MMP14 expression group; yellow: high-MMP14 expression group. **(C)** Correlation of MMP14 expression with immune infiltration levels in DLBCL samples, as assessed by Tumor Immune Estimation Resource (TIMER) analysis.

The abundance ratio of 22 immune cells in the 48 TCGA samples is shown in Figure 4A and Supplementary Figure S1. We found that some immune cells were highly abundant, such as B cells naive, B cells memory, and M0 macrophages (Figure 4A). We further compared the infiltration levels of immune cells between low- and high-MMP14 expression groups. The infiltration levels of B cells naive in the group highly expressing MMP14 were lower compared with those observed in the group expressing low MMP14 levels. However, we observed the opposite situation for M0 and M1 macrophages (Figure 4B). We also found that MMP14 expression had a critically positive correlation with the infiltrating levels of neutrophils and dendritic cells in DLBCL (Figure 4C). Our results suggest that high MMP14 expression levels, other than correlating with a more favorable prognosis, are also associated with high infiltration levels of M0 and M1 macrophages, neutrophils, and dendritic cells. Thus, MMP14 might play an important role in immune infiltration in DLBCL cases.

We further investigated the relationship between MMP14 expression and various immune markers for different immune cells, including monocytes, tumor-associated macrophages (TAMs), M0, M1, and M2 macrophages, neutrophils, and dendritic cells using TIMER and GEPIA (Table 2). After the correlation adjustment based on tumor purity, results showed that the MMP14 expression level remarkably correlated with most immune markers. Notably, results revealed that the expression levels of monocyte, M0, and M1 macrophages showed a strong correlation with MMP14 expression in TIMER (*P* < 0.05; Table 2). Especially M0 macrophages results were similar to those obtained using the GEPIA database (Supplementary Table S4). Important correlation was also found among MMP14 and M0, M2 macrophage markers (Table 2). Collectively, these findings suggested that MMP14 may regulate macrophage cells in DLBCL. Thus, these results further upheld the findings that MMP14 had a correlation with immune-infiltrating cells, especially in macrophage cells or M0 macrophages in DLBCL.

**TABLE 2:** Correlation analysis between MMP14 and immune cell-related genes and markers, as assessed with Tumor Immune Estimation Resource (TIMER).

Description	Gene markers	DLBCL			
		No adjustment		Purity adjusted	
		Cor	P	Cor	P
B cell	CD19	-0.254	8.19e-02	−0.259	1.02e-01
	CD79A	−0.147	3.18e-01	−0.161	3.15e-01
Monocyte	CD86	0.253	8.24e-02	0.319	4.20e-02
	CD115 (CSF1R)	0.636	2.2e-06	0.589	5.07e-05
TAM	CCL2	0.281	5.32e-02	0.265	9.43e-02
	CD68	0.782	0e-00	0.786	1.18e-09
	IL10	0.359	1.26e-02	0.276	8.05e-02
Macrophage M0	MMP9	0.716	5.92e-08	0.66	2.73e-06
	NCF2	0.601	9.70e-08	0.556	1.59e-04
	ACP5	0.633	2.50e-06	0.558	1.49e-04
	PLA2G7	0.687	2.27e-07	0.668	1.85e-06
Macrophage M1	INOS (NOS2)	0.292	4.44e-02	0.181	2.57e-01
	IRF5	0.197	1.79e-01	0.037	8.16e-01
	COX2(PTGS2)	0.246	9.2e-02	0.061	3.14e-01
Macrophage M2	CD163	0.465	9.84e-04	0.488	1.21e-03
	VSIG4	0.502	8.36e-04	0.532	3.48e-04
	MS4A4A	0.281	5.3e-02	0.253	1.10e-01
Neutrophils	CD66b (CEACAM8)	0.252	8.35e-02	0.223	1.61e-01
	CD11b (ITGAM)	0.765	0e-00	0.765	5.55e-09
	CCR7	0.018	9.05e-01	−0.141	3.79e-01
Dendritic cells	HLA-DPB1	0.047	7.48e-01	0.058	7.16e-01
	HLA-DQB1	−0.038	7.95e-01	0.055	7.33e-01
	HLA-DRA	0.117	4.25e-01	0.072	6.55e-01
	HLA-DPA1	0.132	3.72e-01	0.101	5.30e-01
	BDCA-1(CD1C)	−0.153	2.97e-01	−0.203	2.03e-01
	BDCA-4(NRP1)	0.536	1.13e-04	0.421	6.15e-03
	CD11c (ITGAX)	0.443	1.79e-03	0.345	2.72e-02
CD8 + T cell	CD8A	0.205	1.62e-01	0.061	7.05e-01
	CD8B	0.125	3.95e-01	0.021	8.94e-01
T cell (general)	CD3D	−0.022	8.8e-01	0.282	7.37e-02
	CD3E	0.115	4.36e-01	−0.065	6.85e-01
	CD2	0.166	2.58e-01	0.031	8.48e-01
Natural killer cell	KIR2DL1	0.048	7.48e-01	−0.1	5.33e-01
	KIR2DL3	0.15	3.08e-01	0.063	6.96e-01
	KIR2DL4	0.324	2.48e-02	0.204	2.02e-01
	KIR3DL1	0.087	5.58e-01	0.056	7028e-01
	KIR3DL2	0.047	7.49e-01	−0.062	7.00e-01
	KIR3DL3	-0.117	4.27e-01	−0.112	4.87e-01
	KIR2DS4	0.09	5.44e-01	0.013	9.37e-01
Th1	T-bet (TBX21)	0.233	1.11e-01	0.14	3.81e-01
	STAT4	0.16	2.76e-01	-0.052	7.45e-01
	STAT1	0.472	8.19e-04	0.442	3.85e-03
	IFN-γ (IFNG)	0.532	2.15e-02	0.291	6.52e-02
	TNF-α (TNF)	0.338	1.92e-02	0.265	9.38e-02
	GATA3	0.183	2.13e-01	0.035	8.27e-01
	STAT6	0.146	3.2e-01	0.076	6.39e-01
	STAT5A	0.224	1.26e-01	0.225	1.58e-01
	IL13	0.067	9.63e-01	−0.026	8.58e-01
Tfh	BCL6	-0.134	3.61e-01	-0.033	8.38e-01
	IL21	0.167	2.58e-01	0.072	6.57e-01
Th17	STAT3	0.402	4.89e-03	0.315	4.48e-02
	IL17A	0.081	5.83e-01	0.024	8.82e-01

*TAM, tumor-associated macrophage; Cor, R value of Spearman’s correlation; No adjustment, correlation without adjustment. Purity adjusted, and correlation adjusted by purity.*

### Identifying the Immune Cell Most Related to Survival

**FIGURE 5:**
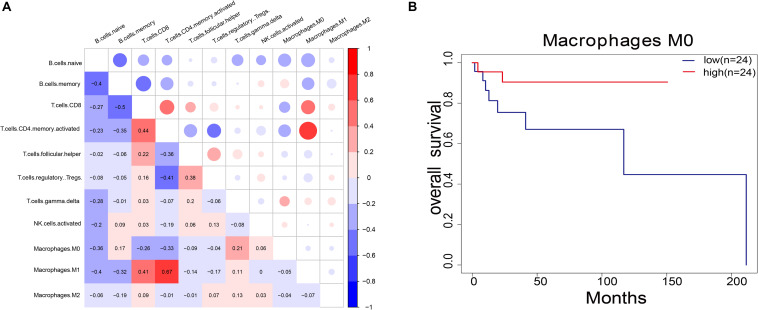
**(A)** Pairwise relationship between immune cell abundance ratios. The numerical values represent the correlation value. Red represents a positive correlation; blue represents a negative correlation. **(B)** Survival analysis of DLBCL patients according to the abundance ratio of M0 macrophages (*P* = 0.08). The red line indicates the high expression group, and the blue line indicates the low expression group.

For the 11 immune cells in the 48 samples, the abundance ratio and their correlation are shown in Figure 5A and their *P-*value in Supplementary Table S5. T-cell CD4 memory activated was significantly associated with M1 macrophage, while B-cell memory showed a negative correlation with T-cell CD4 and T-cell CD8 memory activated. Furthermore, we investigated the relationship between the abundance ratio of 11 types of immune cells and overall patient survival. Results showed that the abundance ratio of M0 macrophages positively correlated to survival (Figure 5B and Supplementary Table S6). This result is consistent with the correlation between MMP14 expression and survival (Figures 2B–E).

### Correlation Between Clinical Characteristics, Macrophages M0, and Its Associated Genes

**FIGURE 6:**
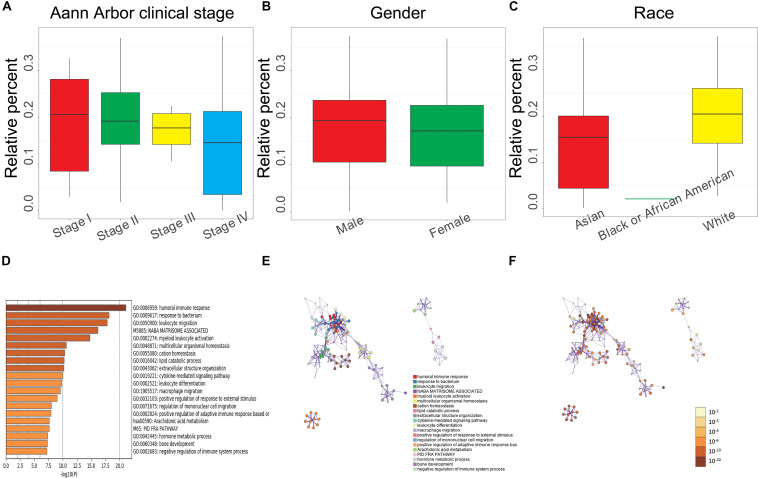
**(A)** Relationship between the M0 macrophage abundance ratios and Ann Arbor clinical stage (Stage I, 8 samples; Stage II, 17; Stage III, 5; and Stage IV, 12; *P* = 0.692). **(B)** Relationship between M0 macrophage abundance ratios and gender (male, 22 samples; female, 26; *P* = 0.344). **(C)** Relationship between M0 macrophage abundance ratios and race (Asian, 18 samples; Black or African American, 1; White, 29; *P* = 0.043). **(A–C)** Each boxplot shows the median with the 25th and 75th percentiles. **(D)** Bar graph of enriched terms based on M0 macrophage abundance levels, colored by *P-*value. **(E)** Enriched terms network. Nodes are colored by cluster-ID and closeness to each other. **(F)** Enriched terms network. Nodes are colored by *P-*value.

To better understand the relationship of the clinical characteristics to M0 macrophages, we performed a correlation analysis between the M0 macrophage abundance ratio and the following clinical characteristics: Ann Arbor clinical stage, gender, and race. We observed that the M0 macrophage abundance ratio was negatively correlated to the Ann Arbor clinical stage, and the abundance ratio in males was higher than that in female patients (Figures 6A,B). We found a significant difference regarding race, with White patients showing a higher abundance ratio than that observed for Black, African American, and Asian patients (Figure 6C), which is consistent with the results obtained for MMP14 expression (Supplementary Table S2).

We also analyzed the correlation between gene differential expression and M0 macrophages abundance and found a total of 398 upregulated (including MMP14) and 194 downregulated genes significantly associated with M0 macrophage abundance. We performed GO and KEGG enrichment analysis using these differential expression genes. The top 20 clusters and their enriched representative terms are shown in Figure 6D. Each node in the network represents an enriched term, colored according to the cluster-ID (Figure 6E) and by its *P-*value (Figure 6F). Results showed enrichment in genes involved in the humoral immune response, macrophage migration, positive regulation of adaptive immune response based on somatic recombination of immune receptors, negative regulation of immune system process, and leukocyte migration.

## Discussion

Recent immunotherapy breakthroughs have shown a novel strategy to effectively treat tumors, and lots of clinical studies are aimed at improving prognosis via novel combinations of immunotherapy and chemotherapy for DLBCL patients (31). The discovery of reliable and robust biomarkers, in addition to the pathological and clinical factors already in use, is required to improve the personalized therapy for patients.

Thus, immunological molecular markers should be strongly considered in the evaluation and treatment of cancer patients. The MMP14 matrix metalloproteinase is a pericellular type I collagenase (32), and previous studies demonstrated that MMP14 acts on the surroundings by promoting metastasis and tissue remodeling (33). However, before this study, the underlying roles of MMP14 in tumor immunology and its suitability as a prognostic biomarker for DLBCL were not investigated.

Here, we reported that the MMP14 expression level was correlated with favorable OS and PFS using independent datasets in the GEO database (GSE98588, GSE10846, and GSE4475; Figures 2B–E), and MMP14 expression was higher in DLBCL tumors than in normal tissues (Figure 2A), also with race in clinical characteristic. These results reached a conclusion that MMP14 was a prognostic biomarker in DLBCL. To date, diverse immune cell infiltration of MMP14 in DLBCL is unclear. Our results demonstrated that M0 macrophages might play a role in DLBCL. First, the abundance of M0 macrophages was high in DLBCL (Figure 4A). The MMP14 expression level was remarkably correlated with most immune markers, especially in regulating M0 macrophages and M2 macrophages (Figure 4 and Table 2). Furthermore, M0 macrophages were similar to those in the GEPIA database (Supplementary Table S4). Second, GO term and KEGG pathway analyses also indicated that the pathways regulating macrophage migration were important in DLBCL patients and strongly related with MMP14 expression level in the TCGA-DLBCL databases (Figure 3). These findings showed that MMP14 might have a role in the regulation of infiltrating immune cells, specifically in macrophage cells in DLBCL. Furthermore, M0 macrophages were the immune cells that were most related to survival (Supplementary Table S3). The low abundance ratio of M0 macrophage patients had an increasing risk of death, which is consistent with the relationship between MMP14 expression and survival (Supplementary Figure S2). Moreover, we found that the abundance ratio of M0 macrophages decreased with the increase in the Ann Arbor clinical stage (Figure 6A). Significant race difference was found and that the White race was the highest (Figures 6B,C), according to the relationship between MMP14 expression and race (Supplementary Table S2). Enrichment analysis also showed that the M0 macrophages were related to the immune system process and leukocyte migration (Figures 6D,E). All these results supported that MMP14 was related to M0 macrophages.

Although little is known about macrophages’ function in tumor immune response in the context of DLBCL, one of the histological hallmarks of B-cell lymphomas is the high number of macrophages involved in the clearance of apoptotic and tumor cells (34, 35). Macrophages are extensively involved in the regulation of immune response and homeostasis, acting as sentinels of the tissue microenvironment (36). Under different stimuli, primary macrophages (M0) can be polarized into either M1 (classically activated) or M2 (alternatively activated) macrophages (37). Activated M1 macrophages might favor peritoneal fibrosis by producing various pro-inflammatory cytokines that cause tissue damage, while M2 mediates inflammation resolution and repair promotion (37). In our study, we discovered that in DLBCL, MMP14 expression is positively correlated to M0 macrophages. We found that M0 macrophage abundance is most closely correlated with survival, and it is negatively correlated with the Ann Arbor clinical stage (Figure 6A). Our findings are consistent with the known role of MMP14, which is upregulated in several types of cancer and promote inflammation, angiogenesis, metastasis, and cancer cell invasion (32). At high clinical stages, MMP14 may promote macrophage polarization into M1 and M2, inducing tissue damage, and a systemic inflammatory response in DLBCL patients. Thus, we hypothesize that the inflammatory response and tumor immunity regulated by MMP14 and M0 macrophage density may have a connection with tumor prognosis and progression in DLBCL patients. However, our results were generated with data present in the databases. Thus, further studies need to validate the link between M0 macrophages and MMP14 by using a different approach, such as gene knockout, experiments based on pathological models, and prospective studies.

In conclusion, we found that high MMP14 expression is associated with favorable prognosis and increased immune cell infiltration in DLBCL cases. MMP14 expression potentially contributed to the immune marker set regulation of TAMs cells, especially in macrophage M0. Therefore, MMP14 likely played a role in immune cell infiltration and is a potential novel prognosis biomarker for DLBCL patients.

## Data Availability Statement

The datasets presented in this study can be found in online repositories. The names of the repository/repositories and accession number(s) can be found in the article/Supplementary Material.

## Author Contributions

WH and JS designed the study. CY performed the study and analyzed the data. CY and WH wrote the manuscript. WH and ZG provided expert consultations and clinical suggestions. All authors reviewed the final version of the manuscript.

## Conflict of Interest

The authors declare that the research was conducted in the absence of any commercial or financial relationships that could be construed as a potential conflict of interest.

## Footnotes

1 http://jvenn.toulouse.inra.fr/app/example.html

2 https://www.oncomine.org/resource/login.html

3 http://metascape.org/gp/index.html
