# Supplementary material for: Matrix Metallopeptidase 14: A Candidate Prognostic Biomarker for Diffuse Large B-Cell Lymphoma
Source: Front Oncol. 2020 Aug 20;10:1520. doi: 10.3389/fonc.2020.01520 (PMC7473157; doi:10.3389/fonc.2020.01520)
Supplement: Supplementary file 4 [file Data_Sheet_1.docx]

**SUPPLEMENTARY MATERIAL**

**FIGURE S1 |** The abundance ratio of 22 immune cells in 48 TCGA-DLBCL patients.

**TABLE S1 |** 29 overlapped genes in two independent DLBCL Gene Expression Omnibus (GEO) cohorts (GSE10846, 420 samples; GSE98588,137 samples).

**TABLE S2 |** Patients’ characteristics in the cohort of 48 TCGA-DLBCL patients according to MMP14 expression level.

**TABLE S3 |** Signaling pathways most significantly correlated with MMP14 expression based on their normalized enrichment score (NES) and p-value.

**TABLE S4 |** Correlation analysis between MMP14 and relate genes and markers of Monocyte, macrophage M0, macrophage M1 and macrophage M2 in GEPIA.

**TABLE S5 |** P value of the correlation between the abundance ratios of various immune cells.

**TABLE S6 |** P value of the survival analysis for the abundance ratios of immune cells.

**Table legend**

**TABLE S2 |** Patients’ characteristics in the cohort of 48 TCGA-DLBCL patients according to MMP14 expression level.

| **Clinicopathological characteristic** | **Low-MMP14 group** **(n=24)** | **High-MMP14 group (n=24)** | **P-value** |
| --- | --- | --- | --- |
| Ann Arbor clinical stage |  |  | 0.2231 |
| I | 5 | 3 |  |
| II | 7 | 10 |  |
| III | 2 | 3 |  |
| IV | 7 | 5 |  |
| Gender |  |  | 0.772 |
| Male | 10 | 12 |  |
| Female | 14 | 12 |  |
| Race |  |  | 0.043 |
| Asian | 12 | 6 |  |
| White | 10 | 18 |  |
| Black or African American | 2 | 0 |  |
| BMI | 25.42(16.73-38.61) | 26.39(16.45-46.03) | 0.286 |
| Age | 58.90(28.67-82.89) | 54.70(23.27-78.01) | 0.303 |

**TABLE S4 |** Correlation analysis between MMP14 and relate genes and markers of Monocyte, macrophage M0, macrophage M1 and macrophage M2 in GEPIA.

| **Description** | **Gene markers** | **DLBCL tumor** | |
| --- | --- | --- | --- |
|  |  | **R** | **P-value** |
| B cell | CD19 | -0.24 | 0.097 |
|  | CD79A | -0.14 | 0.35 |
| Monocyte | CD86 | 0.32 | 0.027 |
|  | CD115 (CSF1R) | 0.51 | 0.00026 |
| TAM | CCL2 | 0.28 | 0.056 |
|  | CD68 | 0.59 | 1.2e-05 |
|  | IL10 | -0.0092 | 0.95 |
| Macrophage M0 | MMP9 | 0.66 | 5e-07 |
|  | NCF2 | 0.46 | 0.0012 |
|  | ACP5 | 0.6 | 6.8e-06 |
|  | PLA2G7 | 0.5 | 0.00035 |
| Macrophage M1 | INOS (NOS2) | 0.37 | 0.01 |
|  | IRF5 | 0.19 | 0.19 |
|  | COX2(PTGS2) | 0.33 | 0.024 |
| Macrophage M2 | CD163 | 0.27 | 0.071 |
|  | VSIG4 | 0.25 | 0.095 |
|  | MS4A4A | 0.46 | 0.11 |
| Neutrophils | CD66b (CEACAM8) | 0.082 | 0.58 |
|  | CD11b (ITGAM) | 0.38 | 0.0093 |
|  | CCR7 | 0.21 | 0.16 |
| Dendritic cells | HLA-DPB1 | 0.087 | 0.56 |
|  | HLA-DQB1 | -0.14 | 0.34 |
|  | HLA-DRA | 0.076 | 0.61 |
|  | HLA-DPA1 | 0.27 | 0.071 |
|  | BDCA-1(CD1C) | -0.16 | 0.28 |
|  | BDCA-4(NRP1) | 0.55 | 6.7e-05 |
|  | CD11c (ITGAX) | 0.1 | 0.5 |
| CD8+ T cell | CD8A | 0.035 | 0.82 |
|  | CD8B | 0.003 | 0.84 |
| T cell (general) | CD3D | 0.049 | 0.74 |
|  | CD3E | 0.089 | 0.55 |
|  | CD2 | 0.17 | 0.26 |
| Natural killer cell | KIR2DL1 | -0.061 | 0.68 |
|  | KIR2DL3 | -0.1 | 0.5 |
|  | KIR2DL4 | 0.3 | 0.043 |
|  | KIR3DL1 | 0.14 | 0.37 |
|  | KIR3DL2 | -0.07 | 0.64 |
|  | KIR3DL3 | 0.012 | 0.94 |
|  | KIR2DS4 | -0.11 | 0.47 |
| Th1 | T-bet (TBX21) | 0.28 | 0.059 |
|  | STAT4 | 0.25 | 0.085 |
|  | STAT1 | 0.39 | 0.007 |
|  | IFN-γ (IFNG) | 0.3 | 0.038 |
|  | TNF-α (TNF) | 0.29 | 0.051 |
|  | GATA3 | 0.14 | 0.34 |
|  | STAT6 | 0.18 | 0.22 |
|  | STAT5A | 0.31 | 0.036 |
|  | IL13 | 0.29 | 0.046 |
| Tfh | BCL6 | -0.00065 | 1 |
|  | IL21 | -0.042 | 0.78 |
| Th17 | STAT3 | 0.22 | 0.14 |
|  | IL17A | 0.042 | 0.78 |

**TABLE S6 |** P value of the survival analysis for the abundance ratios of immune cells.

| **Immune cells** | **P-value** |
| --- | --- |
| B cells naive | 0.9 |
| B cells memory | 0.7 |
| T cells CD8 | 0.9 |
| T cells CD4 memory activated | 0.6 |
| T cells follicular helper | 0.09 |
| T cells regulatory (Tregs) | 0.7 |
| T cells gamma delta | 0.7 |
| NK cells activated | 0.4 |
| Macrophages M0 | 0.08 |
| Macrophages M1 | 0.5 |
| Macrophages M2 | 0.3 |
