# Supplementary figures and images for: Matrix Metallopeptidase 14: A Candidate Prognostic Biomarker for Diffuse Large B-Cell Lymphoma
Source: Front Oncol. 2020 Aug 20;10:1520. doi: 10.3389/fonc.2020.01520 (PMC7473157; doi:10.3389/fonc.2020.01520)

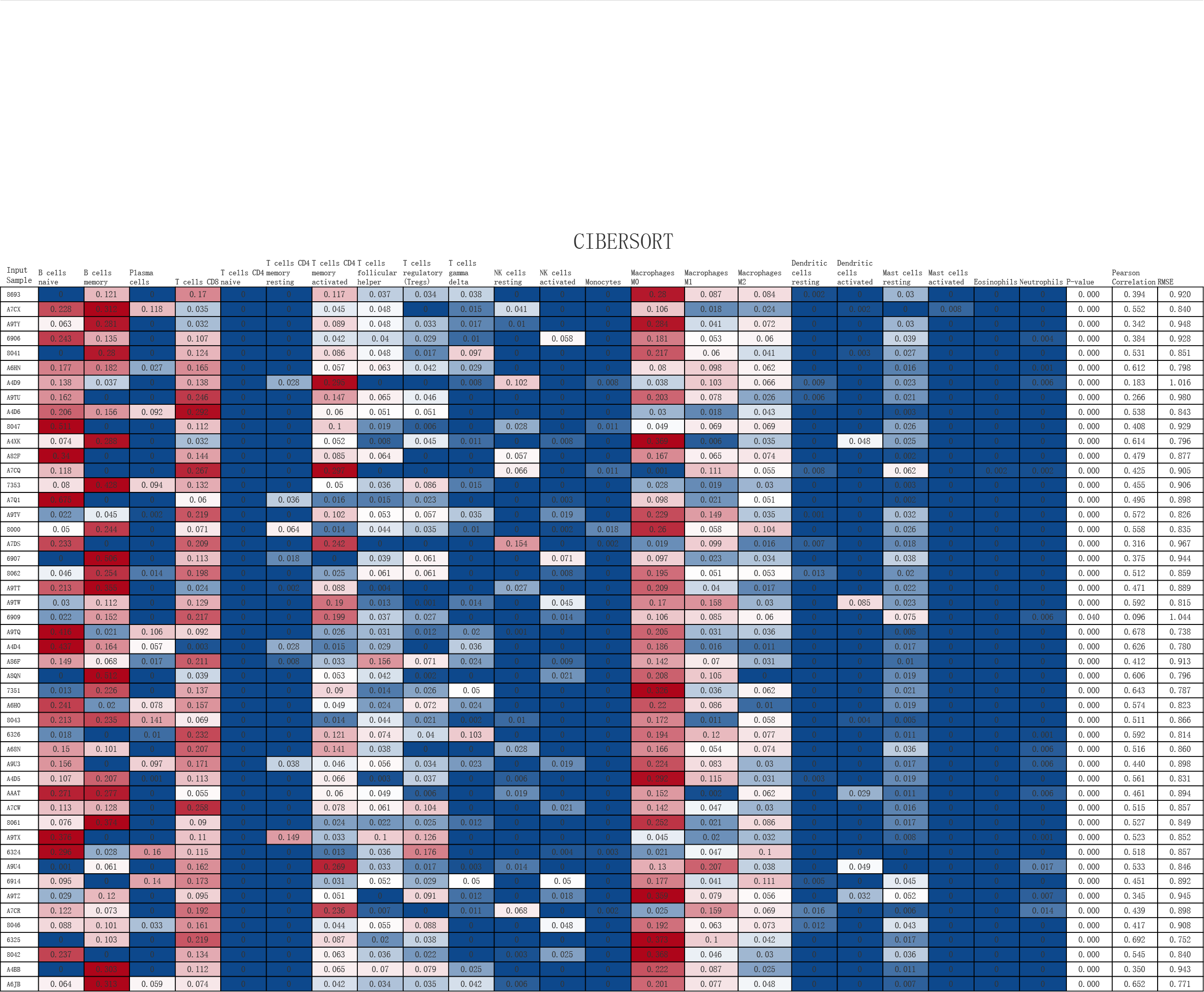

Supplement: Supplementary file 5 [file Image_1.TIF]
